# Case Report: IgG4-Related Disease Presenting With Isolated Hypophysitis

**DOI:** 10.1016/j.aace.2024.07.004

**Published:** 2024-07-15

**Authors:** Suhaib Radi, Michael Tamilia

**Affiliations:** 1 College of Medicine, King Saud Bin Abdulaziz University for Health Sciences, Jeddah, Saudi Arabia; 2 King Abdullah International Medical Research Center, Jeddah, Saudi Arabia; 3 Division of Endocrinology, Department of Internal Medicine, Ministry of the National Guard-Health Affairs, Jeddah, Saudi Arabia; 4 Division of Endocrinology and Metabolism, Jewish General Hospital, McGill University, Montreal, Quebec, Canada

**Keywords:** IgG4-related disease, hypophysitis, pituitary adenoma, pituitary hemorrhage

## Abstract

**Background/Objective:**

IgG4-related disease (IgG4-RD) is an immune-mediated condition that affects multiple organs, including the pituitary gland. Here we present a patient with isolated pituitary involvement of IgG4-RD mimicking pituitary apoplexy.

**Case Report:**

A 49-year-old woman presented to the emergency department with abdominal pain, nausea, vomiting, and weight loss. Her blood pressure was low, and she appeared euvolemic with the rest of physical examination being noncontributory. Her electrolytes showed low serum sodium of 118 mmol/L (normal 135-145). Further investigations were significant for low morning cortisol of 20 nmol/L (N:100-500) and low adrenocorticotropic hormone. Magnetic resonant imaging of the pituitary fossa showed a pituitary macroadenoma with hemorrhagic transformation. She was started on glucocorticoids and levothyroxine before undergoing surgical removal of the pituitary tumor. The pathology was positive for IgG-4-related hypophysitis (IgG4-RH) with no evidence of pituitary tumor.

**Discussion:**

IgG4-RD is an immune-mediated condition that can affect many organs including the pituitary gland, in the form of hypophysitis. IgG4-RH can affect anterior, posterior, or both pituitary lobes. In 2011, Leporati et al developed a diagnostic criteria for IgG4-RH which includes the following: imaging, serology, histopathology, and response t glucocorticoids. The mainstay of treatment is glucocorticoids and hormone replacement therapy.

**Conclusion:**

IgG4-RH might be underestimated and should be suspected in those with hypophysitis or unknown cause of hypopituitarism. Moreover, pituitary macroadenoma with hemorrhagic transformation and panhypopituitarism should be considered as rare and unusual presentations of IgG4-RD.

**Highlights**

• IgG4-related hypophysitis should be suspected in those with hypophysitis or unknown cause of hypopituitarism.
• Affects mostly older men and the majority of patients present with hormonal deficiency ± compressive symptoms.
• Most affected patients will have evidence of systemic IgG4-related disease.
• Diagnosis can be made based on imaging, serum IgG4 level, and biopsy of other involved tissue.
• Mainstay of treatment is glucocorticoid therapy at doses around 40 to 60 mg/d of prednisone.

**Clinical Relevance**

IgG4-related hypophysitis is an underdiagnosed cause of pituitary hormonal insufficiency and can present similar to other causes of hypophyitis and pituitary tumors. Recognition is important as it can be managed medically and avoid invasive neurosurgery.

## Introduction

IgG4-related disease (RD) is an immune-mediated condition comprised of a collection of disorders that share particular features; including tumor-like swelling of involved organs, lymphoplasmacytic infiltrate enriched in IgG4-positive plasma cells, and storiform fibrosis. Multisystemic involvement is usually a feature of this disease, with the following organs/compartments being commonly involved: pancreas, salivary glands, retroperitoneum, and thyroid.^1^

Pituitary gland involvement (hypophysitis) in IgG4-RD has beeno described and commonly misdiagnosed as pituitary tumors. Here we present a patient with isolated pituitary involvement of IgG4-RD mimicking pituitary apoplexy.

## Case Report

A 49-year-old Asian woman, who is known for well-controlled type 2 diabetes mellitus on metformin, bronchial asthma in childhood, and vitiligo, presented to our hospital with a 1 week history of fatigue, nausea, vomiting, headaches, presyncope, and right upper quadrant pain. On further questioning, she reported an 18-month history of similar symptoms with recent worsening over the last 4 months. She had recurrent emergency department visits over the last year with no established diagnosis. When she was seen initially, an abdominal ultrasound was done and revealed a large gallbladder stone of 25 mm with positive sonographic Murphy's sign and possible early acute cholecystitis.

The patient lost 30 pounds over the course of 6 months and reported cold intolerance. She had been menopausal for 9 months. She did not report any galactorrhea, hyperpigmentation, or change in the size of hands or feet. On physical exam, her blood pressure was 92/65 mmHg, heart rate 62 beats per minute, and she was afebrile. Visual field exam by confrontation and eye movements were normal. The rest of her exam was noncontributory.

Her laboratory evaluation revealed low serum sodium at 118 mmol/L (N: 135-145) with serum osmolality of 246 mOsm/kg (N: 280-300). Bicarbonate level was 21 mmol/L (N: 22-26). The rest of her extended electrolytes were normal along with her renal & liver function tests. Urine sodium was 69 mmol/L, and urine osmolality was 275 mOsm/kg. Hormonal profile revealed low morning cortisol at 20 nmol/L (N: 100-500), with low adrenocorticotropic hormone at <0.2 pmol/L (N: 1.6-13.9). Her thyroid-stimulating hormone was also low 0.04 mIU/L (N: 0.4-4), along with low Free thyroxine 8.5 pmol/L (N: 9-26). Estradiol was also low at <18 pmol/L (N: 0-145), with low follicle-stimulating hormone 2.5 U/L (N: 48.6-143.9), and luteinizing hormone 0.4 U/L (N: 13.2-45.7). Insulin-like growth factor 1 was <2.1 nmol (N: 13.4-40.3), and prolactin was elevated at 99.9 mcg/L (N: 3.9-29.5). The summary of her laboratory resulted are in Table 1.

**Table 1:** Laboratory Values at Presentation

Test name	Patient value	Reference range
Serum sodium	118 mmol/L	135-145 mmol/L
Serum osmolality	246 mOsm/kg	280-300 mOsm/kg
Bicarbonate	21 mmol/L	22-26 mmol/L
Potassium	3.7 mmol/L	3.5-5.2 mmol/L
Chloride	96 mmol/L	96-106 mmol/L
Random serum plasma glucose	6 mmol/L	3.9-6.9 mmol/L
Creatinine	63 μmol/L	53- 97 μmol/L
Urea	4.6 mmol/L	2.1- 6.9 mmol/L
Bilirubin	13.1 μmol/L	1.7- 20 μmol/L
ALT	30 IU/L	<41 IU/L
AST	17 IU/L	<40 IU/L
Urine sodium	69 mmol/L	>20 mmol/L
Urine osmolality	275 mOsm/kg	500-850 mOsm/kg
Hemoglobin	13 g/dL	12-16 g/dL
White blood cells	4.6 × 109/L	4-11 × 109/L
Platelets	294 × 109/L	150-450 × 109/L
C-reactive protein	12 mg/L	<5 mg/L
Erythrocyte sedimentation rate	33 mm/h	<20 mm/h
Morning cortisol	20 nmol/L	100-500 nmol/L
ACTH	<0.2 pmol/L	1.6-13.9 pmol/L
TSH	0.04 mIU/L	0.4-4 mIU/L
Free thyroxine	8.5 pmol/L	9-26 pmol/L
Estradiol	<18 pmol/L	0-145 pmol/L
FSH	2.5 U/L	48.6-143.9 U/L
LH	0.4 U/L	13.2-45.7 U/L
IGF-1	<2.1 nmol/L	13.4-40.3 nmol/L
Prolactin	99.9 mcg/L	3.9-29.5 mcg/L

Abbreviations: ACTH = adrenocorticotropic hormone; ALT = alanine aminotransferase; AST = aspartate aminotransferase; FSH = follicle-stimulating hormone; IGF-1 = insulin-like growth factor 1; LH = luteinizing hormone; TSH = thyroid-stimulating hormone.

**Fig. 1:**
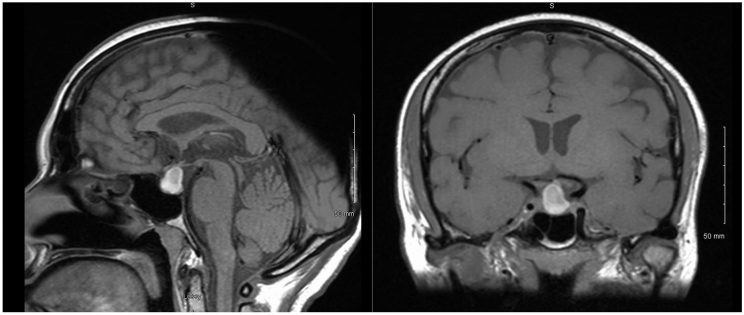
Sagittal and coronal views of T1-weighted images of pituitary magnetic resonance imaging showing an expansile sellar and suprasellar hyperintense lesion measuring 13.5 × 9 × 11 mm.

Because of her presentation, which is consistent with central adrenal insufficiency, central hypothyroidism, hypogonadotropic hypogonadism (low estradiol with inappropriately low follicle-stimulating hormone for her menopausal status), and growth hormone deficiency, she was diagnosed with combined pituitary hormonal deficiency. A magnetic resonance imaging of her pituitary was done and showed an expansile sellar and suprasellar T1-hyperintense lesion with heterogenous signal intensity on T2 and hypointense rim. The lesion measured 13.5 × 9 × 11 mm. It was abutting the left cavernous sinus and causing mild chiasmal and stalk compression. The report’s conclusion was pituitary macroadenoma with hemorrhagic transformation/apoplexy (Fig. 1). It is worth mentioning that although the radiology report was read as “apoplexy.” she did not have the typical clinical presentation that would qualify her as having pituitary apoplexy as it is a clinical diagnosis rather than a radiological one.

**Fig. 2:**
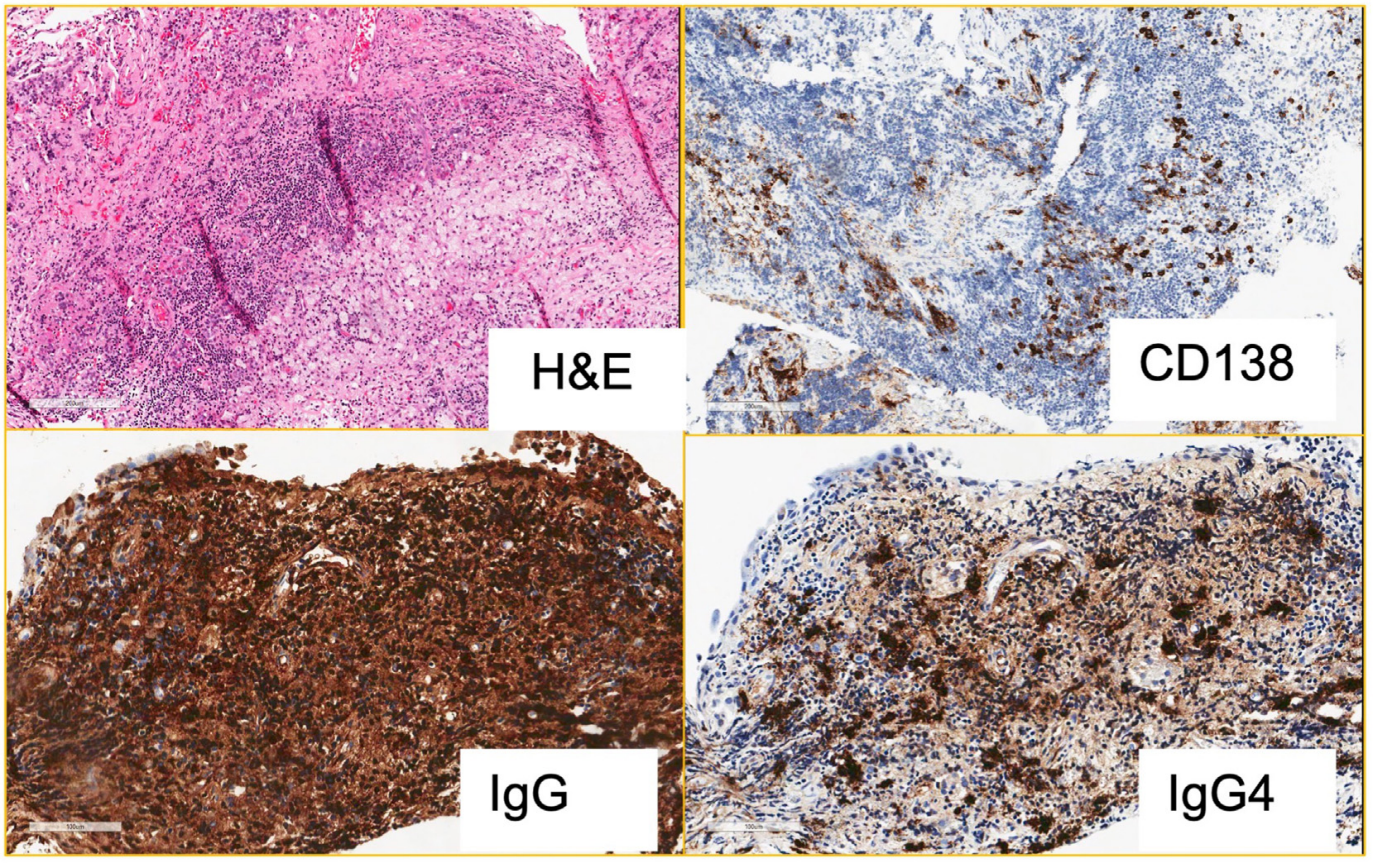
Histopathology of pituitary tumor showing dense inflammatory lymphoplasmacytic infiltrate in the *upper left* picture. The other pictures show immunopositivity for CD 138, IgG, and IgG4. Abbreviation: H&E = hematoxylin & eosin.

Our patient was started on hormone replacement therapy (hydrocortisone and levothyroxine) and neurosurgery was consulted. They elected for surgery given the size of the lesion (>1 cm) that was thought to be a macroadenoma, mass effect on the stalk, and the presence of combined pituitary hormonal deficiency. She underwent transsphenoidal surgical resection of the pituitary tumor. Intraoperatively, the lesion was yellow and avascular with adherent capsule surrounding white opaque fluid. The tumor was distinct from the severely effaced residual pituitary gland. The surgeon was able to completely remove the tumor. To our surprise, the pathology revealed IgG-4-related hypophysitis (RH), with no evidence of an adenoma (Fig. 2). Serum IgG4 level came back high at 2.82 g/L (N: 0.03-2.01) despite being sent 6 weeks after initiating hydrocortisone replacement.

Her postoperative course was uncomplicated, and she is still on her hormonal replacement. She was later assessed by rheumatology and a positron emission tomography scan did not show any evidence of systemic involvement.

## Discussion

IgG4-RD is a fibro-inflammatory condition characterized by a dense lymphoplasmacytic infiltrate rich in IgG4-positive plasma cells, storiform fibrosis, and elevated serum IgG4 concentrations.^2^ It can either affect a single organ or present with multiorgan involvement. Pituitary involvement in IgG4-RD has been described as pituitary hypophysitis.^3^ More recently, apoplexy has been reported in the literature as an atypical uncommon presentation of RH.^4^

In a Japanese review of 170 patients presenting with hypopituitarism and/or central diabetes insipidus at a single center, 7 out of 23 patients with hypophysitis met the diagnostic criteria for IgG4-RH (30%), which constituted 4% out of the total cohort. Four out of those 7 patients had had systemic IgG4-RD.^5^ Another retrospective review of all primary hypophysitis patients who were treated at a German university hospital revealed that 12 out of 29 patients (41%) met criteria for IgG4-RH. This is by no mean a small percentage and they concluded that IgG4-RH should be considered in diagnosis of hypophysitis.^6^

The mean age at presentation is around 60 to 65 years of age with male predominance and the majority of reported cases are of Asian descent.^7^ Usually, more than half of the cases will present with multisystem involvement, with the most commonly affected organs being retroperitoneal fibrosis (26%) and salivary glands (25%).^8^ In those affected with IgG4-RH, the majority (80%-90%) will have 1 or more pituitary hormonal insufficiencies.^5^ In 1 study, 17 out of 18 patients had an element of hypopituitarism. Three patients had hypogonadotropic hypogonadism, 7 had central diabetes insipidus, 3 had central hypothyroidism, 3 had adrenal insufficiency, and 8 patients had panhypopituitarism. Moreover, half the patients had symptoms of mass effect.^7^ Serum IgG4 levels were elevated in two-thirds of the patients and the median level was 264.5 mg/dL (N: 5-105).^5^ In another review of 29 patients with IgG4-RH, 14% of them had isolated pituitary involvement.^5^

Radiological investigations are considered an integral part of the diagnosis. The typical feature on pituitary magnetic resonance imaging is enlargement of the anterior pituitary and the stalk.^5^ Sometimes, IgG4-RH can be mistaken radiologically for other diseases including macroadenoma, Rathke’s cleft cyst, craniopharyngioma, and empty sella.^4^ Although hemorrhagic pituitary lesions are not a typical presentation of hypophysitis, it has been reported in the literature in a young middle-aged woman.^9^ In 2011, Leporati et al developed a diagnostic criteria for IgG4-RH which includes the following: imaging, serology, histopathology, and response to glucocorticoids (Table 2).^3^ Using this criteria, pituitary biopsy was avoided in 75% of patients.^10^

**Table 2:** Diagnostic Criteria for IgG4-Related Hypophysitis∗

Criterion 1	**Pituitary histopathology:**
	mononuclear infiltration of the pituitary gland, rich in lymphocytes and plasma cells, with >10 IgG4-positive cells/high-power field
Criterion 2	**Pituitary MRI:**
	Sellar mass or thickened pituitary stalk
Criterion 3	**Other involvement:**
	Biopsy-proven involvement in other organs
Criterion 4	**Serology:**
	Serum IgG4 level >140 mg/dL (1.4 g/L)
Criterion 5	**Response to treatment:**
	Shrinkage of the pituitary mass and symptom improvement with corticosteroids
Established diagnosis:	
Criterion 1	
Or Criteria 2 + 3	
Or Criteria 2 + 4 + 5	

Abbreviation: MRI = magnetic resonance imaging.

∗ Adapted from reference.^3^

The mainstay of treatment is glucocorticoids and hormone replacement therapy. The glucocorticoid of choice is prednisone at a dose of 0.6 mg/kg/d for 1 to 2 months, followed by a slow taper of 5 mg weekly.^7^ In 1 review, all 18 patients showed clinical improvement after glucocorticoid therapy.^7^ Others reports successful treatment even with physiologic doses of hydrocortisone.^11^ Other therapies that have been successful in glucocorticoid-refractory disease are azathioprine and rituximab.^12^^,^^13^ Interestingly, there is a report of an elderly man with multiple comorbidities, who was diagnosed with IgG4-RH but not started on therapy because of the potential side effects. He was observed for 4 years without steroids or hormonal replacement therapy and his disease has been stable with no progression.^14^

In conclusion, IgG4-RH might be underestimated and should be suspected in those with hypophysitis or unknown cause of hypopituitarism. Moreover, pituitary hemorrhagic transformation with pituitary hormonal insufficiency is a rare presentation of IgG4-RD.

## Disclosure

The authors have no conflicts of interest to disclose.
